# Niraparib with androgen receptor-axis-targeted therapy in patients with metastatic castration-resistant prostate cancer: safety and pharmacokinetic results from a phase 1b study (BEDIVERE)

**DOI:** 10.1007/s00280-021-04249-7

**Published:** 2021-03-22

**Authors:** Fred Saad, Kim N. Chi, Neal D. Shore, Julie N. Graff, Edwin M. Posadas, Jean-Baptiste Lattouf, Byron M. Espina, Eugene Zhu, Alex Yu, Anasuya Hazra, Marc De Meulder, Rao N. V. S. Mamidi, Branislav Bradic, Peter Francis, Vinny Hayreh, Arash Rezazadeh Kalebasty

**Affiliations:** 1 Centre Hospitalier de l’Université de Montréal, Montréal, Canada; 2 BC Cancer, Vancouver, Canada; 3 Carolina Urologic Research Center, Myrtle Beach, SC USA; 4 Knight Cancer Institute, Oregon Health and Science University, Portland, OR USA; 5 Cedars-Sinai Medical Center, Los Angeles, CA USA; 6 Janssen Research & Development, Los Angeles, CA USA; 7 Janssen Research & Development, Raritan, NJ USA; 8 Janssen Research & Development, Spring House, PA USA; 9 Janssen Research & Development, Antwerp, Belgium; 10 Janssen Global Services, Raritan, NJ USA; 11 Norton Cancer Institute, Louisville, KY USA

**Keywords:** PARP inhibitors, Niraparib, Androgen-signaling-targeted therapy, MCRPC, Pharmacokinetics, DRD genes

## Abstract

**Purpose:**

To assess the safety and pharmacokinetics and determine the recommended phase 2 dose (RP2D) of niraparib with apalutamide or abiraterone acetate plus prednisone (AAP) in patients with metastatic castration-resistant prostate cancer (mCRPC).

**Methods:**

BEDIVERE was a multicenter, open-label, phase 1b study of niraparib 200 or 300 mg/day with apalutamide 240 mg or AAP (abiraterone acetate 1000 mg; prednisone 10 mg). Patients with mCRPC were previously treated with ≥ 2 lines of systemic therapy, including ≥ 1 androgen receptor-axis-targeted therapy for prostate cancer.

**Results:**

Thirty-three patients were enrolled (niraparib-apalutamide, 6; niraparib-AAP, 27). No dose-limiting toxicities (DLTs) were reported when combinations included niraparib 200 mg; five patients receiving niraparib 300 mg experienced DLTs [niraparib-apalutamide, 2/3 patients (66.7%); niraparib-AAP, 3/8 patients (37.5%)]. Although data are limited, niraparib exposures were lower when given with apalutamide compared with historical niraparib monotherapy exposures in patients with solid tumors. Because of the higher incidence of DLTs, the niraparib–apalutamide combination and niraparib 300 mg combination with AAP were not further evaluated. Niraparib 200 mg was selected as the RP2D with AAP. Of 19 patients receiving niraparib 200 mg with AAP, 12 (63.2%) had grade 3/4 treatment-emergent adverse events, the most common being thrombocytopenia (26.3%) and hypertension (21.1%). Five patients (26.3%) had adverse events leading to treatment discontinuation.

**Conclusions:**

These results support the choice of niraparib 200 mg as the RP2D with AAP. The niraparib–AAP combination was tolerable in patients with mCRPC, with no new safety signals. An ongoing phase 3 study is further assessing this combination in patients with mCRPC.

**Trial registration no.:**

NCT02924766 (ClinicalTrials.gov).

**Supplementary Information:**

The online version contains supplementary material available at 10.1007/s00280-021-04249-7.

## Introduction

Recent advances in androgen-receptor-axis-targeted therapies (ARAT) for the treatment of metastatic castration-resistant prostate cancer (mCRPC) have led to improved overall survival of up to approximately 35 months in chemotherapy-naive patients and approximately 18 months in patients previously treated with chemotherapy [1–3]. Abiraterone acetate—a prodrug of abiraterone, an androgen biosynthesis inhibitor—in combination with prednisone (AAP), as well as enzalutamide—an androgen receptor inhibitor—showed significant improvements in overall survival and radiographic progression-free survival (rPFS) in patients with mCRPC [2–6]. However, patients who initially respond to ARAT typically develop acquired resistance and eventually undergo relapse [7, 8], indicating a need to develop alternative therapies.

Patients with mCRPC that harbors mutations in homologous recombination repair (HRR) genes (e.g., *BRCA1*/*2*) are sensitive to poly(ADP-ribose) polymerase (PARP) inhibitors [9–11]. The PARP inhibitors niraparib, olaparib, and rucaparib are approved by the US Food and Drug Administration (FDA) for the treatment and maintenance treatment of select patients with ovarian, fallopian tube, and primary peritoneal cancers [12–14]. Another PARP inhibitor, talazoparib, and olaparib are approved by the FDA for the treatment of select patients with breast cancer [13, 15]. Monotherapy with the PARP inhibitors niraparib, olaparib, rucaparib, and talazoparib has recently been shown to improve clinical outcomes in patients with mCRPC and alterations in HRR genes, particularly *BRCA2* [16–21]. Currently, olaparib is approved by the FDA for the treatment of adults with mCRPC and deleterious HRR mutations who progressed after receiving treatment with enzalutamide or abiraterone; recently, rucaparib received accelerated approval for the treatment of adults with mCRPC and deleterious *BRCA* mutation who previously received ARAT and a taxane-based chemotherapy [13, 14]. Although the results of monotherapy with PARP inhibitors are promising, mCRPC continues to be incurable, and additional drugs and drug combinations are needed to expand the existing treatment armamentarium.

In animal studies, HRR genes are shown to interact with androgen receptor signaling, which regulates DNA repair in prostate cancer [22, 23], suggesting that a combination of PARP inhibitors and ARAT may be more effective in patients with HRR mutations. In a randomized phase 2 study that did not select patients based on HRR mutations, the combination of veliparib, a PARP inhibitor, and AAP did not improve the efficacy in patients with mCRPC compared with AAP alone; however, an exploratory analysis revealed a significant association between HRR status and PFS [24]. In another randomized phase 2 trial, the combination of olaparib and AAP was associated with longer median rPFS in patients with mCRPC compared with AAP alone in patients with or without HRR defects [25]; this combination is under further assessment in an ongoing placebo-controlled, randomized, double-blind, phase 3 trial [26]. In another ongoing phase 3 study, the combination of talazoparib and enzalutamide is being investigated as a first-line treatment in patients with mCRPC [27].

As DNA repair is regulated via androgen receptor signaling [22] and that the combination of olaparib and AAP resulted in improved survival in patients with mCRPC [25], we began investigating the combination of niraparib, a potent inhibitor of PARP enzymatic activity and PARP1 trapping, and ARAT (apalutamide or AAP) for the treatment of patients with mCRPC. This phase 1b study was a first step in that direction. The primary objective of the study was to evaluate safety and to establish the recommended phase 2 dose (RP2D) of niraparib when administered with apalutamide or AAP. The secondary objective was to evaluate the pharmacokinetics of niraparib, apalutamide, and abiraterone acetate when administered in the respective combination regimens.

## Methods

### Study population

The BEDIVERE study (ClinicalTrials.gov identifier, NCT02924766) was conducted at five sites in the United States and Canada (see Supplementary Appendix for the list of sites and investigators). Eligible patients were men aged ≥ 18 years with mCRPC with or without HRR mutations. Other inclusion criteria included at least 1 line of prior taxane-based chemotherapy and at least 1 line of prior ARAT for prostate cancer. Exclusion criteria included Eastern Cooperative Oncology Group performance status of ≥ 2, presence of brain metastasis, prior treatment with a PARP inhibitor, and radiotherapy during ≤ 15 days prior to starting treatment. In addition, patients could not have received platelet or red blood cell transfusion, any chemotherapy, hematopoietic growth factors, major surgery, or an investigational product for prostate cancer during 30 days prior to cycle 1 day 1 (C1D1). Patients were required to be castrate via either bilateral orchiectomy or concurrent treatment with a gonadotropin-releasing hormone analog. The full list of inclusion and exclusion criteria is available in the Supplementary Appendix.

### Study design and treatment

**Fig. 1:**
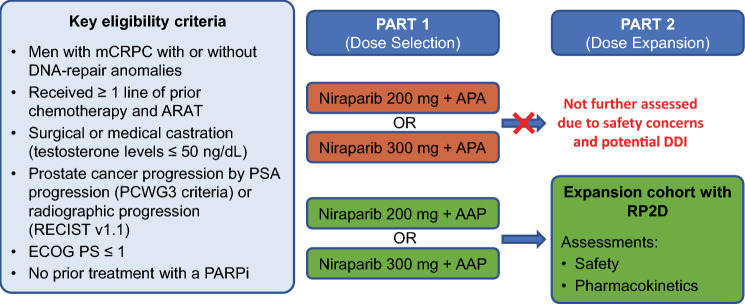
Study design. All patients continued to receive the study treatment until disease progression, unacceptable toxicity, or death. *AAP* abiraterone acetate plus prednisone, *APA* apalutamide, *ARAT* androgen receptor-axis-targeted therapy, *DDI* drug–drug interaction, *ECOG PS* Eastern Cooperative Oncology Group Performance Score, *PARPi* poly(ADP-ribose) polymerase inhibitor, *PCWG3* Prostate Cancer Working Group 3, *PSA* prostate-specific antigen, *RECIST* Response Evaluation Criteria in Solid Tumors, *RP2D* recommended phase 2 dose

BEDIVERE was a 2-part, multicenter, open-label, phase 1b study (Fig. 1). The study was originally designed to assess niraparib in combination with apalutamide to establish niraparib RP2D. However, due to considerable reduction in niraparib exposures due to drug–drug interactions (DDIs) observed with this combination (see below), the study protocol was amended to add the combination of niraparib with AAP, and no further attempts were made to determine a RP2D for the niraparib–apalutamide combination. Part 1 (dose escalation) was a standard 3 + 3 design to determine the RP2D of niraparib when administered with apalutamide or AAP. Part 2 (dose expansion) further assessed safety and pharmacokinetics in up to 15 additional patients for each ARAT. Patients received the combination of niraparib (200 or 300 mg) with either apalutamide (240 mg) or AAP (abiraterone acetate 1000 mg plus prednisone 10 mg); niraparib and apalutamide or AAP were dosed together. All drugs were taken orally once daily, except for prednisone, which was administered 5 mg twice daily. The combination of niraparib and apalutamide was to be taken in the morning with or without food, except on the pharmacokinetics sampling days (see below) when the drugs were taken at the study site after an overnight fast starting at midnight. Niraparib with abiraterone acetate plus the initial daily dose of 5 mg prednisone was to be taken on empty stomach; no food or liquids were to be consumed for at least 2 h before and at least 1 h after dosing.

The RP2D of niraparib was defined separately for the two combinations. The RP2D was determined from safety data as the dose at which fewer than one-third of patients experienced a dose-limiting toxicity (DLT). Additionally, the RP2D could be the same as the clinical dose of niraparib monotherapy (i.e., 300 mg once daily) [12] or, if significant DDIs were observed, a dose with an exposure comparable to or lower than that of historical niraparib 300 mg monotherapy data (i.e., mean maximum plasma concentration [*C*_max_] ranging from 582 to 2230 ng/mL or mean area under the concentration–time curve over the dosing interval [AUC_tau_] ranging from 14,659 to 46,900 ng h/mL [12, 28–30]).

All patients continued to receive the study drugs in 28-day cycles until disease progression, unacceptable toxicity, death, or study termination by the sponsor. Safety monitoring and decisions regarding dose escalation/de-escalation and DLTs were made by a safety-evaluation team that was established by the sponsor and comprised at least one each of medical expert, statistician, and clinical pharmacologist.

### Safety assessments and dose-limiting toxicity

Safety assessments were based on the reported adverse events (AEs) and the results of vital sign measurements, 12-lead electrocardiograms, physical examinations, and clinical laboratory tests. Hypertension, neutropenia, leukopenia, lymphopenia, anemia, and thrombocytopenia were considered AEs of special interest in this study. The DLT assessments were performed during the first treatment cycle (28 days) in Part 1; patients who discontinued treatment during this period due to reasons other than DLT could be replaced. Dose modifications for toxicities were allowed during the DLT-evaluation period. Patients who missed doses for reasons other than toxicities were included in the pharmacokinetics analysis if no more than two consecutive doses were missed, no more than four doses were missed in a cycle, and the last three doses before serial pharmacokinetics sampling were not missed.

The criteria for DLTs were treatment-related grade 4 thrombocytopenia or grade ≥ 3 thrombocytopenia requiring platelet transfusion; treatment-related grade 4 anemia or grade ≥ 3 anemia requiring blood transfusion; treatment-related grade 4 neutropenia for ≥ 7 days or grade ≥ 3 neutropenia with infection or fever > 38.5°C; concurrent elevation of alanine aminotransferase or aspartate aminotransferase > 3 × upper limit of normal and bilirubin > 2 × upper limit of normal (unless the concurrent elevation was related to biliary obstruction or other causes unrelated to study treatment); seizures of any grade, grade 3 fatigue lasting for > 5 days, grade 3 nausea persisting for > 3 days despite treatment, or grade ≥ 3 vomiting or diarrhea persisting for > 3 days despite treatment; grade ≥ 3 hypertension despite > 2 weeks of treatment; any other grade ≥ 3 nonhematologic toxicity except grade 3 rash; or any treatment-related toxicity requiring a dose interruption.

### Pharmacokinetics assessments

Plasma samples were analyzed to determine concentrations and pharmacokinetic properties of niraparib and its major metabolite M1, apalutamide and its metabolite M3 (JNJ-56142060), and abiraterone using validated liquid chromatography with tandem mass spectrometry, with concentrations of 5.0 ng/mL, 25 ng/mL, and 0.2 ng/mL, respectively, as the lower limits of quantification. Additional details of the bioanalytical methods are available in the Supplementary Appendix. Serial blood samples were collected in Parts 1 and 2 on day 1 of cycles 1, 2, and 3, and every 3 cycles thereafter (Supplementary Table 1). The assessed pharmacokinetic parameters included *C*_max_, time to reach *C*_max_ (*t*_max_), AUC over 24 h (AUC_0–24_), and trough plasma concentration (*C*_trough_). Concentration–time profiles were plotted for each analyte, and individual and mean plasma concentration–time data and pharmacokinetic parameters were summarized using descriptive statistics.

### Statistical analyses

This phase 1b study comprised a standard 3 + 3 dose selection (Part 1), followed by a dose expansion at RP2D (Part 2), and the sample size was not formally determined. The sample size of Part 1 was determined by the maximum number of patients required to establish the RP2D of niraparib in combination with ARAT. For Part 2, a sample size of 15 patients was selected based on expected AE rates. The safety population comprised all patients who received ≥ 1 dose of study drug; the pharmacokinetics-evaluable population included all patients who received ≥ 1 dose of study drug and had sufficient and interpretable pharmacokinetic assessments.

All AEs reported during the treatment and follow-up period (30 days after the last dose of study drug) were considered as treatment-emergent AEs (TEAEs), which were summarized by incidence, intensity, type, and relationship to study drug, coded using the Medical Dictionary for Regulatory Activities v21.1, and graded using National Cancer Institute Common Terminology Criteria for Adverse Events v4.03 or higher. Deaths that occurred during treatment or within 30 days after the last dose of study drug were defined as on-treatment deaths.

Pharmacokinetic analyses were performed using the validated Phoenix™ WinNonlin^®^ software (v6.2.1; Tripos LP, USA). Noncompartmental analysis (model, Plasma [200–202]; dose, extravascular) was applied for the pharmacokinetic analysis. The pharmacokinetics outputs were created using SAS v9.3 (SAS Institute Inc., Cary, NC, USA). Summary statistics, including N, mean, SD, % coefficient of variation, geometric mean, median, minimum, and maximum, were calculated for plasma concentration of each analyte at each time point and for the derived pharmacokinetic parameters.

## Results

### Patient disposition and demographic and baseline clinical characteristics

The study was conducted from October 24, 2016, to July 16, 2019. A total of 33 patients were enrolled and treated: 6 in the niraparib–apalutamide group and 27 in the niraparib–AAP group (Supplementary Fig. 1).

In the niraparib–apalutamide group, three patients each received niraparib 200 and 300 mg in Part 1; the median (range) follow-up duration was 15.8 (10.6–22.4) months. Three patients (50%) each discontinued treatment because of progressive disease or AEs. The niraparib–apalutamide group did not advance to Part 2 (see more below).

In the niraparib–AAP group, 4 and 8 patients, respectively, received niraparib 200 and 300 mg in Part 1; an additional 15 patients received niraparib 200 mg in Part 2. The median (range) follow-up duration was 9.0 (0.6–23.9) months. Patients discontinued treatment because of progressive disease (15 patients [55.6%]), AEs (5 [18.5%]), patient withdrawal (4 [14.8%]), physician decision (2 [7.4%]), or death (1 [3.7%]) (Supplementary Fig. 1).

Patient demographics and baseline characteristics were generally similar between the two groups (Table 1). All patients had received ≥ 2 lines of systemic therapy, including ≥ 1 line of ARAT and taxane therapy.

**Table 1:** Demographic and baseline clinical characteristics: Enrolled population

Characteristic	Niraparib + APA	Niraparib + AAP
	Total (*N* = 6)	Total (*N* = 27)
Age, median (range), years	72 (53–81)	68 (49–82)
Race, *n* (%)		
White	6 (100.0)	22 (81.5)
Black	0	3 (11.1)
Other	0	1 (3.7)
Not reported	0	1 (3.7)
ECOG PS, *n* (%)		
0	2 (33.3)	15 (55.6)
1	4 (66.7)	12 (44.4)
PSA, median (range), ng/mL	45.1 (21–1395)	67.1 (3–1230)
Extent of disease progression, *n* (%)		
Bone	6 (100.0)	20 (74.1)
Lymph node	2 (33.3)	13 (48.1)
Liver	0	6 (22.2)
Lung	1 (16.7)	4 (14.8)
Gleason score at initial diagnosis, *n* (%)		
≥ 8	6 (100.0)	17 (70.8)^a^
Prior radiotherapy, *n* (%)	6 (100.0)	27 (100.0)
Prior lines of systemic therapies, *n* (%)^b^	6 (100.0)	27 (100.0)
2	2 (33.3)	16 (59.3)
3	2 (33.3)	8 (29.6)
≥ 4	2 (33.3)	3 (11.1)
Prior lines of ARAT, *n* (%)		
1	3 (50.0)	21 (80.8)^c^
2	2 (33.3)	5 (19.2)^c^
3	1 (16.7)	0
Prior ARAT, *n* (%)		
Enzalutamide	4 (66.7)	19 (70.4)
Abiraterone^d^	3 (50.0)	11 (40.7)
Investigational	3 (50.0)	1 (3.7)
Other^e^	5 (83.3)	25 (92.6)
Prior taxanes, *n* (%)	6 (100.0)	27 (100.0)
Docetaxel	6 (100.0)	25 (92.6)
Cabazitaxel	2 (33.3)	5 (18.5)

*AAP* abiraterone acetate plus prednisone, *APA* apalutamide, *ECOG PS* Eastern Cooperative Oncology Group performance status, *PSA* prostate-specific antigen

^a^*N* = 24

^b^ARAT, taxane, cytotoxic chemotherapy, or other therapy for prostate cancer

^c^*N* = 26

^d^Includes abiraterone and abiraterone acetate

^e^Includes bicalutamide, flutamide, nilutamide, degarelix, and cyproterone acetate

### Treatment exposure and dose adjustments

In the niraparib–apalutamide group (*N* = 6), the median number of treatment cycles administered was 6.0 and 1.0 in the niraparib 200-mg and 300-mg cohorts, respectively (Table 2). The median duration of treatment was 4.7 months in the 200-mg cohort (one patient [33.3%] received treatment for ≥ 6 months) and 0.9 months in the 300-mg cohort; the median relative dose intensity was 99.3% and 85.8%, respectively.

**Table 2:** Treatment exposure and dose adjustments for niraparib. Safety population

Parameter	Niraparib + APA (Part 1)		Niraparib + AAP (Parts 1 + 2)	
	200 mg (*N* = 3)	300 mg (*N* = 3)	200 mg (*N* = 19)	300 mg (*N* = 8)
Treatment exposure				
No. of cycles started, median (range), *n*	6.0 (4.0–7.0)	1.0 (1.0–5.0)	4.0 (1.0–24.0)	4.5 (1.0–12.0)
Treatment duration, median (range), months	4.7 (3.0–6.5)	0.9 (0.9–4.0)	3.7 (0.5–22.0)	3.7 (0.4–11.1)
Relative dose intensity, median (range), %	99.3 (99–100)	85.8 (62–100)	94.6 (31–100)	63.8 (28–100)
Dose reduction, *n* (%)	0	1 (33.3)	4 (21.1)	3 (37.5)
Dose reduced due to TEAE	0	1 (33.3)^a^	4 (21.1)	3 (37.5)
Dose reduced to 200 mg^b^	N/A	1 (33.3)	N/A	2 (25.0)
Dose reduced to 100 mg^b^	0	0	4 (21.1)	1 (12.5)
Dose interruption, *n* (%)	2 (66.7)	2 (66.7)	12 (63.2)	6 (75.0)
Dose interrupted due to TEAE	0^c^	2 (66.7)	12 (63.2)^d^	6 (75.0)
No. of dose interruptions due to TEAE				
1	0	2 (66.7)	6 (31.6)	3 (37.5)
2	0	0	3 (15.8)	2 (25.0)
≥ 3	0	0	3 (15.8)	1 (12.5)
Duration of interruption, median (range), days	1.0 (1–1)	7.5 (7–8)	12.0 (3–48)	9.0 (6–26)

*AAP* abiraterone acetate plus prednisone, *APA* apalutamide, *N/A* not applicable, *TEAE* treatment-emergent adverse event

^a^This patient also had reasons other than TEAE for dose reduction

^b^Patients with > 1 dose reduction are counted only once according to the largest change in the dose level

^c^Both patients in this cohort had reasons other than TEAE for dose interruption

^d^Four patients in this cohort also had reasons other than TEAE for dose interruption

In the niraparib–AAP group (*N* = 27), the median number of treatment cycles administered was 4.0 and 4.5 in the niraparib 200-mg and 300-mg cohorts, respectively. The median duration of treatment was 3.7 months in both cohorts; four patients each in the 200-mg and 300-mg cohorts (21.1% and 50.0%, respectively) received treatment for ≥ 6 months, including one patient each in the 200-mg and 300-mg cohorts who received treatment for ≥ 21 and ≥ 11 months, respectively. Additional data on dose reductions and interruptions in each cohort are shown in Table 2.

### Safety assessments

In the niraparib–apalutamide group, no DLTs were reported in the niraparib 200-mg cohort; in the 300-mg cohort, two patients (66.7%) experienced DLTs: one patient (33.3%) had grade 4 thrombocytopenia, and another (33.3%) had grade 3 fatigue and grade 3 hypertension. This combination was not further assessed in Part 2 (see below).

In the niraparib–AAP group, no DLTs were reported in the niraparib 200-mg cohort. In the niraparib 300-mg cohort, one patient (12.5%) had 2 DLTs: grade 4 elevated gamma-glutamyl transferase and grade 3 fatigue; two additional patients (25.0%) had grade 4 neutropenia at C2D1 (after the DLT-evaluation period), which were also considered as DLTs. Considering that 37.5% of patients (3 of 8) in this cohort had DLTs, which is above the acceptable DLT limit of 33% further assess a drug or combination, the niraparib 300 mg in combination with AAP was not further assessed. Niraparib 200 mg was selected as the RP2D in combination with AAP and was further evaluated in 15 additional patients in Part 2.

In the niraparib–apalutamide group, grade 3/4 TEAEs were reported in two (66.7%) and three patients (100.0%) in the 200-mg and 300-mg cohorts, respectively (Table 3). At least one TEAE leading to study drug discontinuation was reported in one patient (33.3%) in the 200-mg cohort (ventricular dyskinesia and ventricular extrasystole) and two patients (66.7%) in the 300-mg cohort [thrombocytopenia and hypertension (one patient each)]. All six patients in this group died due to progressive disease; however, none of the deaths occurred during treatment, and no deaths were attributed to TEAEs.

**Table 3:** Safety results. Safety population

*n* (%)	Niraparib + APA (Part 1)		Niraparib + AAP (Parts 1 + 2)	
	200 mg (*N* = 3)	300 mg (*N* = 3)	200 mg (*N* = 19)	300 mg (*N* = 8)
Safety summary				
≥ 1 TEAE	3 (100.0)	3 (100.0)	19 (100.0)	8 (100.0)
Related TEAE^a^	3 (100.0)	3 (100.0)	19 (100.0)	6 (75.0)
≥ 1 serious TEAE	1 (33.3)	1 (33.3)	4 (21.1)	4 (50.0)
Related serious TEAE	1 (33.3)	1 (33.3)	2 (10.5)	0
Dose-limiting toxicities	0	2 (66.7)	0	3 (37.5)
≥ 1 grade 3/4 TEAE	2 (66.7)	3 (100.0)	12 (63.2)	7 (87.5)
≥ 1 TEAE leading to study drug discontinuation^b^	1 (33.3)	2 (66.7)	5 (26.3)	2 (25.0)
≥ 1 TEAEs leading to death^b^	0	0	1 (5.3)^c^	2 (25.0)^d^
Most common grade 3/4 TEAEs^e^				
Fatigue	1 (33.3)	2 (66.7)	1 (5.3)	1 (12.5)
Arthralgia	2 (66.7)	0	1 (5.3)	0
Thrombocytopenia	0	1 (33.3)	5 (26.3)	0
Hypertension	0	1 (33.3)	4 (21.1)	1 (12.5)
Anemia	0	0	2 (10.5)	2 (25.0)
Neutropenia	0	0	1 (5.3)	2 (25.0)
General physical health deterioration	0	0	1 (5.3)	2 (25.0)
Back pain	0	0	0	2 (25.0)
Sepsis	0	0	0	2 (25.0)
Nausea	0	0	3 (15.8)	1 (12.5)
Vomiting	0	0	3 (15.8)	0
Blood phosphorus decreased	0	0	2 (10.5)	0
TEAEs of special interest^f^	1 (33.3)	2 (66.7)	13 (68.4)	3 (37.5)
Anemia	0	1 (33.3)	5 (26.3)	3 (37.5)
Leukopenia	1 (33.3)	1 (33.3)	2 (10.5)	0
Hypertension	1 (33.3)	1 (33.3)	6 (31.6)	2 (25.0)
Thrombocytopenia	0	1 (33.3)	6 (31.6)	1 (12.5)
Neutropenia	0	1 (33.3)	2 (10.5)	2 (25.0)
Lymphopenia	0	0	1 (5.3)	0

*AAP* abiraterone acetate plus prednisone, *APA* apalutamide, *TEAE* treatment-emergent adverse event

^a^Assessed by investigator as possibly, probably, or likely related to study treatment

^b^Included grade 5 events

^c^Patient had serious grade 3 deterioration in general physical health, which was considered by the investigator as unrelated to niraparib or AAP; see Supplementary Appendix for additional details

^d^One patient had serious grade 3 TEAE of deterioration in general physical health (considered by the investigator as unrelated to niraparib or AAP) and progressive disease; another patient had serious grade 3 deterioration in general physical health (considered by the investigator as unrelated to niraparib or AAP); see Supplementary Appendix for additional details

^e^Occurring in > 1 patient in any cohort; arranged by descending incidence in any cohort

^f^Any grade; 1 patient each had grade 3 congestive heart failure and myocardial infarction, but they were not deemed to be TEAEs of special interest

In the niraparib–AAP group, grade 3/4 TEAEs were reported in 12 (63.1%) and 7 patients (87.5%) in the 200-mg and 300-mg cohorts, respectively. At least one TEAE leading to study drug discontinuation was reported in five patients (26.3%) in the 200-mg cohort (nausea and vomiting [two patients], elevated gamma-glutamyl transferase, thrombocytopenia, and congestive heart failure [one patient each]) and two patients (25.0%) in the 300-mg cohort (fatigue and back pain [one patient each]). In the 200-mg cohort, 12 patients (63.2%) died during the study—11 due to progressive disease and 1 due to TEAE (general physical health deterioration)—and 1 patient died due to disease progression 192 days after discontinuing the study. In the 300-mg cohort, two patients (25.0%) died during treatment or within 30 days after the last dose of any study drug: one due to progressive disease and TEAE (deterioration of general physical health) and one due to TEAE (deterioration of general physical health). None of the three deaths due to TEAEs that occurred during the study were considered related to any study drug by the investigators; the detailed narratives of these patients are provided in the Supplementary Appendix.

At the RP2D of niraparib (200 mg) with AAP (*N* = 19), the most common grade 3/4 AEs were thrombocytopenia (26.3%), hypertension (21.1%), nausea (15.8%), vomiting (15.8%), anemia (10.5%), and hypophosphatemia (10.5%) (Table 3). The most common any-grade TEAEs of special interest were thrombocytopenia (31.6%), hypertension (31.6%), anemia (26.3%), leukopenia (10.5%), and neutropenia (10.5%). The incidence of TEAEs of special interest is reported in Table 3.

### Pharmacokinetics of niraparib and its metabolite M1 (niraparib–apalutamide group)

The mean *C*_max_ (315 ng/mL) of niraparib 200 mg when administered with apalutamide was achieved in a median of 3.0 h postdose at C1D28, with a biphasic decline thereafter; the mean AUC_0–24_ was 4388 ng h/mL (Supplementary Fig. 2; Supplementary Table 2). The pharmacokinetics results for niraparib 300 mg were available for only two patients in this group and, therefore, summary statistics were not calculated. At C1D28, the *C*_max_ values for these two patients were 842 and 820 ng/mL and AUC_0–24_ values were 15,097 and 13,607 ng h/mL (Supplementary Table 2), which were below or at the lower end of the range for the historical monotherapy data for niraparib in solid tumors (*C*_max_ range, 582–2230 ng/mL; AUC_0–tau_ range, 14,659–46,900 ng h/mL [12, 28–30]). This could suggest a potential DDI resulting in reduced exposures of niraparib when administered with apalutamide (see more below). Because niraparib 300 mg in combination with apalutamide resulted in DLTs in 66.7% of patients and grade 3/4 TEAEs despite a reduced niraparib exposure, this combination was not further assessed in Part 2.

The mean *C*_max_ (702 ng/mL) of M1 in patients who received niraparib 200 mg with apalutamide was achieved in a median of 6.0 h postdose at C1D28 and declined steadily thereafter (Supplementary Fig. 2; Supplementary Table 2). The M1 concentrations were markedly greater than those of niraparib, with the metabolite-to-parent ratio of 3.25. The pharmacokinetics results of M1 in 2 patients who received niraparib 300 mg with apalutamide are reported in Supplementary Table 2.

### Pharmacokinetics of niraparib and its metabolite M1 (niraparib—AAP group)

**Fig. 2:**
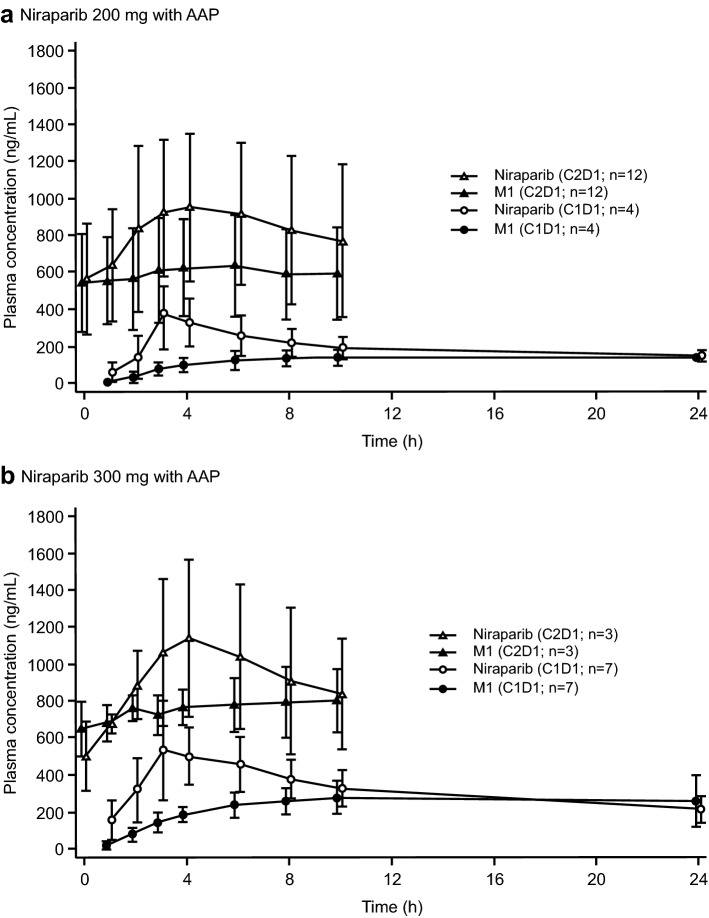
Plasma concentration–time profile of niraparib and its metabolite M1 at C1D1 and C2D1 when niraparib 200 mg (**a**) or niraparib 300 mg (**b**) was administered with AAP once daily: Pharmacokinetics-evaluable population. AAP abiraterone acetate 1000 mg with prednisone 10 mg, *C* cycle, *D* day

When niraparib was administered with AAP, the shape of the niraparib concentration–time profiles was similar between C1D1 and C2D1 with expected accumulation at C2D1 compared to C1D1 (Fig. 2). The niraparib *C*_max_ was achieved at a median of approximately 3 h postdose at C1D1 and at 4 h postdose at C2D1 (Table 4). The mean *C*_max_ values at C2D1 were approximately twofold greater compared with C1D1; as expected, the values were greater with niraparib 300 mg vs 200 mg dose. The trends for mean AUC_0–24_ were generally similar, with greater values for C2D1 vs C1D1 and for niraparib 300 mg vs 200 mg, although the difference between mean AUC_0–24_ with niraparib 200 and 300 mg was relatively small at C2D1. The mean *C*_trough_ values at C2D1 for niraparib 200 and 300 mg were comparable. The niraparib *C*_max_ (1141 ng/mL) and AUC_0–24_ (18,536 ng h/mL) values at C2D1 with niraparib 300 mg coadministered with AAP were comparable to the historical niraparib monotherapy data (*C*_max_ range 582–2230 ng/mL; AUC_0–tau_ range 14,659–46,900 ng h/mL [12, 28–30]).

**Table 4:** Pharmacokinetic parameters of niraparib and abiraterone when niraparib was administered with AAP. Pharmacokinetics-evaluable population

Pharmacokinetic Parameter	Niraparib 200 mg + AAP		Niraparib 300 mg + AAP	
	C1D1 (*N* = 4)	C2D1 (*N* = 11)	C1D1 (*N* = 7)	C2D1 (*N* = 3)
Niraparib				
*C*_max_, mean (SD), ng/mL	379 (194)	985 (409)	589 (232)	1141 (426)
*t*_max_, median (range), h	3.26 (3.00–4.00)	4.00 (2.00–6.35)	3.00 (2.98–6.00)	4.00 (4.00–4.02)
AUC_0–24_, mean (SD), ng h/mL	5139 (1629)^a^	17,745 (9380)^b,c^	7527 (2421)^d^	18,536 (6512)^c^
*C*_trough_, mean (SD), ng/mL	N/A	564 (299)^e^	N/A	505 (188)
M1				
*C*_max_, mean (SD), ng/mL	143 (47.7)	625 (254)	283 (115)	830 (156)
*t*_max_, median (range), h	10.00 (5.98–10.52)	4.07 (1.92–10.35)	10.00 (6.08–24.00)	6.00 (2.02–8.00)
AUC_0–24_, mean (SD), ng h/mL	3187 (505)^a^	13,549 (6141)^b,c^	5551 (2080)^d^	17,728 (354)^c^
*C*_trough_, mean (SD), ng/mL	N/A	545 (265)^e^	N/A	650 (148)
M1:niraparib AUC_0-24_ ratio, mean (SD)	0.64 (0.97)^a^	0.88 (0.42)^b^	0.75 (0.17)^d^	0.99 (0.16)
Abiraterone				
*C*_max_, mean (SD), ng/mL	NA	137 (69.4)^b^	NA	53.4; 83.2^f^
*t*_max_, median (range), h	NA	1.35 (1.00–2.00)^b^	NA	2.00; 3.00^f^
AUC_0–24_, mean (SD), ng h/mL	NA	712 (140)^b,c^	NA	313; 363^f^
*C*_trough_, mean (SD), ng/mL	NA	9.67 (5.32)^e^	NA	5.44 (0.95)

*AAP* abiraterone acetate plus prednisone, *AUC*_*0–24*_ area under concentration–time curve from 0 to 24 h, *C* cycle, *C*_*max*_ maximum plasma concentration, *C*_*trough*_ trough plasma concentration, *D* day, *N/A* not applicable, *NA* not available, *t*_*max*_ time to *C*_max_

^a^*N* = 3

^b^*N* = 10

^c^Predose concentration used for calculation at 24 h

^d^*N* = 6

^e^*N* = 13

^f^Data for one patient was not assessable; individual data for two patients are provided

The concentration–time profiles of M1 were also generally similar in shape at both 200-mg and 300-mg doses at C1D1 and C2D1 (Fig. 2). The M1 *C*_max_ was achieved at a median of 10 h postdose at C1D1 and at approximately 4–6 h postdose at C2D1 (Table 4). The mean *C*_max_ values of M1 were markedly greater at C2D1 vs C1D1; as expected, the values were greater with niraparib 300 mg vs 200 mg. The trends for mean AUC_0–24_ were generally similar, with greater values at C2D1 vs C1D1 (due to accumulation) and with niraparib 300 mg vs 200 mg. The *C*_trough_ values at C2D1 for niraparib 200-mg and 300-mg doses were comparable. The metabolite-to-parent ratios were below 1 in all cohorts.

### Pharmacokinetics of apalutamide and its metabolite M3 (niraparib–apalutamide group)

The mean *C*_max_ of apalutamide (5.15 µg/mL) in patients who received niraparib 200 mg with apalutamide was achieved in a median of 3.0 h postdose at C1D28, declined slightly, and remained nearly constant thereafter; the mean AUC_0-24_ was 92.4 µg h/mL (Supplementary Fig. 3; Supplementary Table 3). The concentration of the apalutamide metabolite M3 remained nearly constant through 24 h postdose; the mean *C*_max_ (5.29 µg/mL) was achieved at 24.0 h postdose at C1D28. The mean AUC_0–24_ of M3 was 112 µg h/mL, and the metabolite-to-parent AUC_0–24_ ratio was 1.26. The pharmacokinetic characteristics of apalutamide and M3 in patients who received niraparib 300 mg with apalutamide were not summarized, because data were available for only two patients (Supplementary Table 3). These results were consistent with apalutamide monotherapy exposures in patients with prostate cancer [31].

### Pharmacokinetics of abiraterone (niraparib–AAP group)

The abiraterone *C*_max_ in patients who received niraparib 200 mg with AAP was achieved in a median of 1.35 h postdose at C2D1 and declined steadily thereafter; the mean AUC_0–24_ was 712 ng h/mL (Table 4; Supplementary Fig. 4). The mean *C*_trough_ levels of abiraterone at C2D1 were slightly lower for niraparib 300 mg vs niraparib 200 mg. The summary statistics for abiraterone *C*_max_, *t*_max_, and AUC_0–24_ in patients who received niraparib 300 mg with AAP could not be calculated, because data were available for only two patients (Table 4).

## Discussion

BEDIVERE, a 2-part, open-label, phase 1b study was designed primarily to assess safety of niraparib 200 and 300 mg in combination with apalutamide or AAP and to determine the RP2D of niraparib in patients with mCRPC who had been previously treated for prostate cancer. The secondary objective of the study was to assess pharmacokinetics of niraparib, apalutamide, and abiraterone acetate. No patient experienced DLTs in Part 1 with niraparib 200 mg in combination with either apalutamide or AAP; however, treatment with niraparib 300 mg led to DLTs in 66.7% and 37.5% of patients when combined with apalutamide and AAP, respectively. Grade 3/4 fatigue occurred in a greater proportion of patients in the niraparib–apalutamide group (niraparib 200 mg, 33.3%; niraparib 300 mg, 66.7%) compared with those in niraparib–AAP group (5.3% and 12.5%, respectively), suggesting that apalutamide was the probable cause of fatigue in patients in those cohorts. Furthermore, because coadministration of apalutamide with niraparib 300 mg was associated with greater toxicity and reduced niraparib exposure (hypothesized to be caused by DDI; see below), niraparib 200 mg with apalutamide was not assessed further. Niraparib 200 mg was selected as the RP2D in combination with AAP. Because no DLTs were reported with niraparib 200 plus AAP in Part 1, the dose-expansion cohort (Part 2) of 15 additional patients further assessed and acquired additional pharmacokinetics and safety data on this combination.

The niraparib RP2D of 200 mg with AAP was well tolerated, with a safety profile consistent with the single agents. The median relative dose intensity for niraparib was 94.6% during a median treatment period of 3.7 months in this cohort. Niraparib 200 mg in combination with AAP is currently being assessed in an ongoing randomized, placebo-controlled, double-blind, phase 3 study (MAGNITUDE; NCT03748641) in patients with mCRPC, regardless of an alteration in a HRR gene.

Although the sample sizes in the present study were relatively small, a few potential explanations were considered for the reduced niraparib exposure when given in combination with apalutamide. Since apalutamide is a pregnane X receptor (PXR) inducer, one potential explanation is that apalutamide induces metabolism of niraparib, leading to its reduced exposure. To evaluate whether apalutamide could induce the carboxylesterase pathway, niraparib clearance and M1 formation were compared in an in vitro metabolism study in apalutamide-induced and non-induced human hepatocytes. As niraparib clearance and M1 formation were not increased in apalutamide-induced hepatocytes (unpublished data), induction via the M1 pathway is unlikely to explain the reduced niraparib exposure when administered with apalutamide. Another possibility is that niraparib exposure was reduced due to induction of P-glycoprotein (P-gp) by apalutamide, given that niraparib is a P-gp substrate [12]. Rifampin, which, like apalutamide, is a potent PXR inducer, has shown up to a 67% decrease in exposure of various P-gp substrates [32]. In a recent study, treatment with apalutamide resulted in a 30% reduction in AUC_0–last_ of fexofenadine, also a P-gp substrate, in patients with CRPC; the reduction was considered to be related to PXR-mediated induction of P-gp and inhibition of P-gp in the gut by apalutamide [33]. Although P-gp induction by apalutamide may not completely explain the extent of observed interaction, a minor contribution of this mechanism cannot be ruled out. It is plausible that both variability in pharmacokinetics (limited data; no niraparib-alone arm) and P-gp induction by apalutamide contributed to the observed reduced niraparib exposure when combined with apalutamide. The exposures of apalutamide and its metabolite were consistent with apalutamide monotherapy exposures in patients with prostate cancer [31].

The steady-state *C*_max_ and AUC_0–24_ values for niraparib 200 or 300 mg in combination with AAP at C2D1 were within the RP2D target exposure range, suggesting absence of a DDI with this combination. Niraparib exposure, when used in combination with AAP, was dose proportional, as reported previously for niraparib monotherapy; the time to maximum concentration was also similar (approximately 3–4 h) [29]. The abiraterone pharmacokinetics for the combination were also comparable to abiraterone 1000 mg monotherapy data [34].

### Study limitations

This was a phase 1b study with a relatively small sample size, and a larger phase 3 study is needed to further confirm the findings of this signal-finding study. The exact mechanism underlying the reduced niraparib exposure when it is combined with apalutamide is not well understood and will require further investigation. Although the combination of niraparib 200 mg and apalutamide was tolerable, the combination was not further assessed in Part 2. Similarly, a reduced dose of apalutamide with niraparib 300 mg, which could possibly resolve the toxicity and potential DDI with this combination, was not assessed.

## Conclusions

In this multicenter, open-label, phase 1b study, patients with mCRPC received treatment with niraparib 200 or 300 mg in combination with either apalutamide or AAP. In both groups, DLTs were observed in some patients who received niraparib 300 mg. In addition, although sample size was limited, pharmacokinetics data suggested a reduced niraparib exposure when niraparib 300 mg was administered with apalutamide; no such change in niraparib exposure was observed when niraparib was administered with AAP. Therefore, the niraparib–apalutamide combination is not being pursued for further evaluation. Based on an acceptable safety profile and comparable exposure to the clinically effective niraparib monotherapy dose, niraparib 200 mg was selected as the RP2D in combination with AAP. The combination of niraparib 200 mg with AAP was tolerable in patients with mCRPC, with no new safety signals; the combination is currently being studied in a randomized, placebo-controlled, phase 3 study in patients with mCRPC, regardless of HRR mutations.

## Supplementary Information

Below is the link to the electronic supplementary material.

Supplementary file1 (DOCX 187 KB)

## Data Availability

The data sharing policy of Janssen Pharmaceutical Companies of Johnson & Johnson is available at https://www.janssen.com/clinical-trials/transparency. As noted on this site, requests for access to the study data can be submitted through Yale Open Data Access (YODA) Project site at http://yoda.yale.edu.
