# Supplementary material for: Niraparib with androgen receptor-axis-targeted therapy in patients with metastatic castration-resistant prostate cancer: safety and pharmacokinetic results from a phase 1b study (BEDIVERE)
Source: Cancer Chemother Pharmacol. 2021 Mar 22;88(1):25–37. doi: 10.1007/s00280-021-04249-7 (PMC8149334; doi:10.1007/s00280-021-04249-7)
Supplement: Supplementary file 1 — Supplementary file1 (DOCX 187 KB) [file 280_2021_4249_MOESM1_ESM.docx]

**Niraparib With Androgen Receptor-Axis–Targeted Therapy in Patients With Metastatic Castration-Resistant Prostate Cancer: Safety and Pharmacokinetic Results From A Phase 1b Study (BEDIVERE)**

Fred Saad^1^, Kim N. Chi^2^, Neal D. Shore^3^, Julie N. Graff^4^, Edwin M. Posadas^5^, Jean-Baptiste Lattouf^1^, Byron M. Espina^6^, Eugene Zhu^7^, Alex Yu^8^, Anasuya Hazra^8^, Marc De Meulder^9^, Rao N. V. S. Mamidi^7^, Branislav Bradic^7^, Peter Francis^10^, Vinny Hayreh^6^, Arash Rezazadeh Kalebasty^11^

^1^Centre Hospitalier de l’Université de Montréal, Montréal, Canada; ^2^BC Cancer, Vancouver, Canada; ^3^Carolina Urologic Research Center, Myrtle Beach, SC, USA; ^4^Knight Cancer Institute, Oregon Health and Science University, Portland, OR, USA; ^5^Cedars-Sinai Medical Center, Los Angeles, CA, USA; ^6^Janssen Research & Development, Los Angeles, CA, USA; ^7^Janssen Research & Development, Raritan, NJ, USA; ^8^Janssen Research & Development, Spring House, PA, USA; ^9^Janssen Research & Development, Antwerp, Belgium; ^10^Janssen Global Services, Raritan, NJ, USA; ^11^Norton Cancer Institute, Louisville, KY, USA

**SUPPLEMENTARY APPENDIX**

**Table of Contents**

[List of sites and investigators 2](#_Toc61425366)

[Full list of inclusion and exclusion criteria 3](#_Toc61425367)

[Inclusion criteria 3](#_Toc61425368)

[Exclusion criteria 4](#_Toc61425369)

[Bioanalytical methods 7](#_Toc61425370)

[Abiraterone 7](#_Toc61425371)

[Apalutamide and metabolite M3 (JNJ-56142060) 7](#_Toc61425372)

[Niraparib and M1 metabolite 7](#_Toc61425373)

[Narratives of patients with TEAEs leading to death 8](#_Toc61425374)

[References 9](#_Toc61425375)

[Table S1 Blood sample collection schedule for pharmacokinetics assessments 10](#_Toc61425376)

[Table S2 Pharmacokinetic characteristics of niraparib and its metabolite M1 when niraparib was administered with apalutamide (C1D28): pharmacokinetics-evaluable population 11](#_Toc61425377)

[Table S3 Pharmacokinetic characteristics of apalutamide and its metabolite M3 when niraparib was administered with apalutamide (C1D28): pharmacokinetics-evaluable population 12](#_Toc61425378)

[Fig. S1 Patient treatment disposition. Enrolled population 13](#_Toc61425379)

[Fig. S2 Plasma concentration-time profile of niraparib and its metabolite M1 at C1D28 when niraparib 200 mg was administered with apalutamide 240 mg once daily: pharmacokinetics-evaluable population 14](#_Toc61425380)

[Fig. S3 Plasma concentration-time profile of apalutamide and its metabolite M3 at C1D28 when niraparib 200 mg was administered with apalutamide 240 mg once daily: pharmacokinetics-evaluable population 15](#_Toc61425381)

[Fig. S4 Plasma concentration-time profile of abiraterone at C2D1 when niraparib 200 or 300 mg was administered with AAP once daily: pharmacokinetics-evaluable population 16](#_Toc61425382)

# List of sites and investigators

| **Site** | **Address** | **Principal Investigator** |
| --- | --- | --- |
| **Canada** |  |  |
| Site 1 | Centre Hospitalier de l’Université de Montréal, Montréal | Fred Saad |
| Site 2 | BC Cancer Agency, Vancouver | Kim N. Chi |
| **USA** |  |  |
| Site 1 | Carolina Urologic Research Center, Myrtle Beach, SC | Neal Shore |
| Site 2 | Knight Cancer Institute, Oregon Health and Science University, Portland, OR | Julie N. Graff |
| Site 3 | Cedars-Sinai Medical Center, Los Angeles, CA | Edwin M. Posadas |

# Full list of inclusion and exclusion criteria

## Inclusion criteria

- Male
- ≥ 18 years of age (or higher legal age of consent in the jurisdiction in which the study is took place)
- Signed informed consent form
- Diagnosis of prostate adenocarcinoma as confirmed by the investigator (mixed histology is acceptable, with the exception of the small cell pure type phenotype, which is excluded) (criterion modified per Amendment 2)
- At least 1 line of prior taxane-based chemotherapy
- At least 1 line of prior androgen-receptor–targeted therapy for prostate cancer; however, patients previously treated with enzalutamide, abiraterone acetate, or apalutamide were considered for this study only if no other treatment options were available (criterion modified per Amendment 2)
- Progression of metastatic prostate cancer in the setting of castrate levels of testosterone ≤ 50 ng/dL on a gonadotropin releasing hormone analog (GnRHa), or history of bilateral orchiectomy at study entry defined as having one or more of the following (criterion modified per Amendment 2):
  - Prostate-specific antigen (PSA) progression defined by a minimum of 2 rising PSA levels with an interval of ≥ 1 week between each determination (per Prostate Cancer Working Group 3 [PCWG3] criteria) [1]; The PSA value at the screening visit should be ≥ 2 μg/L
  - Radiographic progression of soft tissue by Response Evaluation Criteria in Solid Tumors (RECIST) v1.1 or bone disease by PCWG3 criteria [1]. as defined below:
    - Soft tissue disease (measurable) by RECIST v1.1 criteria defined as having nodal disease (pelvic or extrapelvic [retroperitoneal, mediastinal, thoracic, other]) with lesions ≥ 1.5 cm in the short axis and/or visceral disease (lung, liver, adrenal) with lesions ≥ 1 cm in the long axis
    - Bone disease (non-measurable) defined as having bone lesions in the absence of measurable soft tissue disease
- Must be able to continue GnRHa during the course of the study if not surgically castrate (criterion modified per Amendment 2)
- Eastern Cooperative Oncology Group performance status of ≤ 1
- Must be able to swallow whole study drugs
- Patient must agree to use medically accepted and highly effective methods of contraception during the course of the study and for 3 months after the last dose of study drug
- To avoid risk of drug exposure through the ejaculate (even men with vasectomies), patients must agree while on study drug and for 3 months following the last dose of study drug to use a condom during sexual activity and not donate sperm
- At screening, the following laboratory parameters must be met (criterion modified per Amendment 2):
  - Absolute neutrophil count ≥ 1.5 x 10^9^/L
  - Hemoglobin ≥ 9.0 g/dL
  - Platelet count ≥ 100 x 10^9^/L
  - Serum albumin ≥ 3 g/dL
  - Serum creatinine ≤ 1.5 × upper limit of normal (ULN), or a calculated creatinine clearance ≥ 60 mL/min using the Cockcroft-Gault equation
  - Serum potassium ≥ 3.5 mmol/L
  - Serum total bilirubin ≤ 2.5 × ULN or direct bilirubin ≤ 1 x ULN; patients with Gilbert’s syndrome with total bilirubin > 1.5 × ULN and direct bilirubin ≤ 1.5 × ULN could be eligible
  - Aspartate aminotransferase or alanine aminotransferase ≤ 2.5 × ULN (or ≤ 5 x ULN in the presence of liver metastases)
  - Circulating tumor cells count of ≥ 1 cells/7.5 mL blood
- Must be willing to provide a tumor tissue sample (archival or recently collected), if feasible), and a blood sample (criterion modified per Amendment 2)

## Exclusion criteria

- Known brain metastases
- Prior treatment with a poly(ADP-ribose) polymerase (PARP) inhibitor
- Known history or current diagnosis of myelodysplastic syndrome or acute myeloid leukemia
- Known symptomatic or impending cord compression, except if patient has received definitive treatment for this and demonstrates evidence of clinically stable disease
- Active non-prostate cancer (except for basal cell or squamous cell skin cancer, non-muscle invasive bladder cancer [stages pTaG1 and pTaG2], or any other cancer in situ currently in complete remission) within 2 years prior to cycle 1 day 1 (C1D1)
- History of seizure or condition that may predispose to seizure (including, but not limited to prior stroke, transient ischemic attack, or loss of consciousness ≤ 1 year prior to enrollment; brain arteriovenous malformation; or intracranial masses such as schwannomas and meningiomas that are causing edema or mass effect)
- Current or prior treatment with antiepileptic medications for the treatment of seizures
- Known allergies, hypersensitivity, or intolerance to niraparib, apalutamide, or abiraterone acetate or the excipients of niraparib, apalutamide, or abiraterone acetate (criterion modified per Amendment 2) [2]
- Any condition for which, in the opinion of the investigator, participation would not be in the best interest of the patient (eg, compromise the well-being) or that could prevent, limit, or confound the protocol-specified assessments
- Known disorder affecting gastrointestinal absorption
- Prior radiotherapy ≤ 15 days prior to C1D1, with the exception of a single fraction of radiotherapy for the purposes of palliation, which was permitted
- History of clinically significant ventricular arrhythmias (eg, ventricular tachycardia, ventricular fibrillation, Torsades de pointes)
- Prolonged corrected QT interval by the Fridericia correction formula (QTcF) on the screening electrocardiogram > 450 msec (criterion modified per Amendment 2)
- Receiving concomitant medications known to prolong QTc, or which are associated with Torsades de pointes, and patients are unable to discontinue use while receiving study drug
- History or evidence for any of the following: severe or unstable angina or myocardial infarction, symptomatic congestive heart failure, arterial or venous thromboembolic events (eg, pulmonary embolism, cerebrovascular accident including transient ischemic attacks) within 6 months prior to C1D1 or New York Heart Association Class III to IV heart disease
- Presence of uncontrolled hypertension (systolic blood pressure [BP] > 160 mmHg or diastolic BP > 100 mmHg). Patients with a history of hypertension are allowed, provided that BP is controlled to within these limits by anti-hypertensive treatment
- Patient is an employee of the investigator or study site, with direct involvement in the proposed study or other studies under the direction of that investigator or study site, as well as family members of the employees or the investigator
- Human immunodeficiency virus-positive patients with 1 or more of the following:
  - Not receiving highly active antiretroviral therapy
  - A change in antiretroviral therapy within 6 months of the start of screening (except if, after consultation with the sponsor on exclusion criterion 18c, a change is made to avoid a potential DDI with the study drug)
  - Receiving antiretroviral therapy that may interfere with the study drug (consult the sponsor for review of medication prior to enrollment)
  - CD4 count < 350 at screening
  - An acquired immunodeficiency syndrome-defining opportunistic infection within 6 months of the start of screening
- Received or had ≤ 30 days prior to C1D1:
  - a transfusion (platelets or red blood cells)
  - chemotherapy
  - hematopoietic growth factors
  - an investigational agent for prostate cancer
  - major surgery
- Prior platinum-based chemotherapy for the treatment of prostate cancer (criterion added per Amendment 2)
- Left ventricular ejection fraction of < 50% as determined by multiple uptake gated acquisition or echocardiography during screening

# Bioanalytical methods

## Abiraterone

Samples were processed using liquid/liquid extraction with a sample volume of 100 μL. Separation was achieved by high-performance liquid chromatography (HPLC) that used a Waters Acquity BEH C18 column (50 × 2.1 mm, 1.7 μm) at 50°C and 0.1% formic acid in water as mobile phase A and methanol:acetonitrile (50:50 v/v) as mobile phase B, operating at a gradient with an initial flow rate of 0.600 mL/min. A Sciex API4000 triple quadrupole mass spectrometer equipped with a turbo ion spray source was used for detection in positive ion mode. Quantification was based on multiple reaction monitoring (MRM) of the transitions of m/z 350.2 – 156.0 for abiraterone and m/z 354.2 – 160.0 for internal standard abiraterone-d4. A calibration curve ranging from 0.200 to 500 ng/mL was plotted.

## Apalutamide and metabolite M3 (JNJ-56142060)

Samples were processed using protein precipitation extraction with a sample volume of 25 μL. Separation was achieved by HPLC that used a Waters Acquity XBridge C18 column (50 × 2.1 mm, 3.5 μm) at 60°C and 0.1% formic acid in water as mobile phase A and acetonitrile as mobile phase B, operating at a gradient with an initial flow rate of 0.500 mL/min. A Sciex API5000 triple quadrupole mass spectrometer equipped with a turbo ion spray source was used for detection in negative ion mode. Quantification was based on MRM of the transitions of m/z 476.1 – 419.1 for apalutamide, m/z 464.1 – 235.0 for M3 metabolite, m/z 479.1 – 419.1 for internal standard apalutamide-d3, and m/z 469.2 – 240.1 for internal standard metabolite-d4. A calibration curve ranging from 25.0 to 25000 ng/mL was plotted.

## Niraparib and M1 metabolite

Samples were processed using protein precipitation extraction with a sample volume of 25 μL. Separation was achieved by HPLC that used a Waters Acquity BEH C18 column (50 × 2.1 mm, 1.7 μm) at 55°C and 0.5% acetic acid in water as mobile phase A and methanol as mobile phase B, operating at a gradient with an initial flow rate of 0.400 mL/min. A Sciex 5500 triple quadrupole mass spectrometer equipped with a turbo ion spray source was used for detection in positive ion mode. Quantification was based on MRM of the transitions of m/z 321.0 – 304.0 for Niraparib, m/z 322.0 – 304.0 for M1 metabolite, m/z 328.0 – 311.0 for internal standard niraparib-d7, and m/z 329.0 – 311.0 for internal standard metabolite-d7. A calibration curve ranging from 5.00 to 2500 ng/mL was plotted.

# Narratives of patients with TEAEs leading to death

A total of 3 patients had TEAEs leading to death. One patient who received niraparib 200 mg and AAP had nonserious grade 3 thrombocytopenia on day 28, which was probably related to niraparib; patient withdrew consent on day 31, and treatment was permanently discontinued. The TEAE of thrombocytopenia was resolved by day 40. A serious grade 3 TEAE of deterioration in general physical health was reported on day 45; patient was transferred to palliative care on day 46 and subsequently died on day 49 due to deterioration in general physical health, which was considered by the investigator as unrelated to niraparib or AAP

One patient who had ongoing grade 4 elevated gamma-glutamyl transferase and grade 2 elevated aspartate aminotransferase from day −14, received niraparib 300 mg and abiraterone acetate from day 1 to 7 and prednisone from day 1 to 13. This patient experienced serious grade 3 TEAE of deterioration in general physical health, which was considered by the investigator as unrelated to niraparib or AAP, on day 16 and subsequently died on day 17 due to this TEAE and progressive disease. Another patient who received niraparib 300 mg and AAP had on day 26 grade 3 TEAEs of serious deterioration in general physical health and nonserious small intestinal obstruction, which were considered by the investigator as not related to niraparib or AAP. On day 29, nonserious grade 3 TEAEs of dyspnea, pneumonia, respiratory failure, and urinary tract infection were reported, none of which were considered as related to study drugs. Patient had grade 3 acute renal failure on day 31 and died on day 33 due to deterioration in general physical health, which was considered as not related to the study drugs. Patient had received the last dose of niraparib and abiraterone acetate on day 24 and the last dose of prednisone on day 33.

# References

1. Scher HI, Morris MJ, Stadler WM, Higano C, Basch E, Fizazi K, Antonarakis ES, Beer TM, Carducci MA, Chi KN, Corn PG, de Bono JS, Dreicer R, George DJ, Heath EI, Hussain M, Kelly WK, Liu G, Logothetis C, Nanus D, Stein MN, Rathkopf DE, Slovin SF, Ryan CJ, Sartor O, Small EJ, Smith MR, Sternberg CN, Taplin ME, Wilding G, Nelson PS, Schwartz LH, Halabi S, Kantoff PW, Armstrong AJ, Prostate Cancer Clinical Trials Working G (2016) Trial design and objectives for castration-resistant prostate cancer: updated recommendations from the Prostate Cancer Clinical Trials Working Group 3. J Clin Oncol 34 (12):1402-1418. doi:10.1200/JCO.2015.64.2702

2. Jones P, Wilcoxen K, Rowley M, Toniatti C (2015) Niraparib: a poly(ADP-ribose) polymerase (PARP) inhibitor for the treatment of tumors with defective homologous recombination. J Med Chem 58 (8):3302-3314. doi:10.1021/jm5018237

# Table S1 Blood sample collection schedule for pharmacokinetics assessments

| **Blood Sample For** | **C1D1** | | | | | | | | **C2D1** | | | | | | | | **C3D1^a^** | **D1 Every 3 Cycles^a^** |
| --- | --- | --- | --- | --- | --- | --- | --- | --- | --- | --- | --- | --- | --- | --- | --- | --- | --- | --- |
|  | **Sampling Time in Relation to Dosing (Hour)** | | | | | | | | | | | | | | | | | |
|  | **1** | **2** | **3** | **4** | **6** | **8** | **10** | **24^b,c^** | **−1^b^** | **1** | **2** | **3** | **4** | **6** | **8** | **10** | **−1^b^** | **−1^b^** |
| Part 1 (dose escalation) | | | | | | | | | | | | | | | | | | |
| Niraparib and M1 | X | X | X | X | X | X | X | X | X | X | X | X | X | X | X | X | X | X |
| APA, M3, and abiraterone |  |  |  |  |  |  |  |  | X | X | X | X | X | X | X | X | X | X |
| Part 2 (dose expansion)^b^ | | | | | | | | | | | | | | | | | | |
| Niraparib and M1 |  |  |  |  |  |  |  |  | X | X | X | X | X | X | X | X | X | X |
| Abiraterone^d^ |  |  |  |  |  |  |  |  | X |  |  |  |  |  |  |  | X | X |
| APA apalutamide, C cycle, D day  ^a^Trough samples  ^b^Samples collected within 60 minutes prior to study drug administration  ^c^Day 2  ^d^Blood samples for APA and its metabolite M3 were planned but not collected because treatment with niraparib and APA did not proceed to Part 2 | | | | | | | | | | | | | | | | | | |

# Table S2 Pharmacokinetic characteristics of niraparib and its metabolite M1 when niraparib was administered with apalutamide (C1D28): pharmacokinetics-evaluable population

| **Pharmacokinetic** **Characteristic** | **Niraparib + APA** | |
| --- | --- | --- |
|  | **200 mg**  **(N = 3)** | **300 mg**  **(N = 2)**^a^ |
| Niraparib |  |  |
| C_max_, mean (SD), ng/mL | 315 (58.0) | 842, 820 |
| t_max_, median (range), h | 3.00 (3.00–3.02) | 4.00, 2.00 |
| AUC_0-24_, mean (SD), ng•h/mL | 4388 (519) | 15097, 13607 |
| C_trough_, mean (SD), ng/mL | 148 (33.0) | 511, 607 |
| M1 |  |  |
| C_max_, mean (SD), ng/mL | 702 (196) | 1030, 2030 |
| t_max_, median (range), h | 6.00 (4.00–6.00) | 6.00, 2.00 |
| AUC_0-24_, mean (SD), ng•h/mL | 13797 (4314) | 20385, 43416 |
| C_trough_, mean (SD), ng/mL | 482 (110) | 726, 1810 |
| M1:niraparib AUC_0-24_ ratio, mean (SD) | 3.25 (1.42) | 1.35, 3.19 |
| APA apalutamide, AUC_0-24_ area under concentration-time curve from 0 to 24 hours, C cycle, C_max_ maximum plasma concentration, C_trough_ trough plasma concentration, D day, t_max_ time to C_max_  ^a^Data for 1 patient was not assessable; individual data for 2 patients are provided. | | |

# Table S3 Pharmacokinetic characteristics of apalutamide and its metabolite M3 when niraparib was administered with apalutamide (C1D28): pharmacokinetics-evaluable population

| **Pharmacokinetic Characteristic** | **Niraparib + Apalutamide** | |
| --- | --- | --- |
|  | **200 mg**  **(N = 3)** | **300 mg**  **(N = 3)^a^** |
| Apalutamide |  |  |
| C_max_, mean (SD), µg/mL | 5.15 (0.70) | 8.58; 7.29 |
| t_max_, median (range), h | 3.00 (1.00–3.00) | 2.00; 2.00 |
| AUC_0-24_, mean (SD), µg•h/mL | 92.4 (23.6) | 146; 129 |
| C_trough_, mean (SD), µg/mL | 3.57 (0.51) | 5.28; 6.45 |
| M3 |  |  |
| C_max_, mean (SD), µg/mL | 5.29 (1.13) | 5.40; 5.80 |
| t_max_, median (range), h | 24.00 (1.00–24.00) | 3.00; 0.00 |
| AUC_0-24_, mean (SD), µg•h/mL | 112 (25.7) | 112; 115 |
| C_trough_, mean (SD), µg/mL | 4.69 (0.785) | 4.48; 5.80 |
| M3:APA AUC_0-24_ ratio, mean (SD), | 1.26 (0.05) | 0.77; 0.89 |
| APA apalutamide, AUC_0-24_ area under concentration-time curve from 0 to 24 hours, C cycle, C_max_ maximum plasma concentration, C_trough_ trough plasma concentration, D day, t_max_ time to C_max_  ^a^Data for 1 patient was not assessable; individual data for 2 patients are provided. | | |

# Fig. S1 Patient treatment disposition. Enrolled population

**Discontinued Treatment**

n = 3

Progressive disease, 2 (67%)

Adverse event, 1 (33%)

**Niraparib 200 mg + APA**

n = 3

**Niraparib 200 mg + AAP**

n = 4

**Discontinued Treatment**

n = 3

Adverse event, 2 (67%)

Progressive disease, 1 (33%)

**Niraparib 300 mg + APA**

n = 3

**Discontinued Treatment**

n = 19

Progressive disease, 10 (53%)

Adverse event, 4 (21%)

Patient withdrawal, 3 (16%)

Physician decision, 2 (11%)

**Niraparib 200 mg + APA**

n = 15

**Discontinued Treatment**

n = 8

Progressive disease, 5 (63%)

Adverse event, 1 (13%)

Patient withdrawal, 1 (13%)

Death, 1 (13%)

**Niraparib 300 mg + AAP**

n = 8

**Enrolled**

N = 33

**Niraparib + APA**

n = 6

**Niraparib + AAP**

n = 27

**Part 1**

Dose Selection

**Part 2**Expansion

All patients continued to receive the study treatment until disease progression, unacceptable toxicity, death, or study termination by the sponsor.

AAP abiraterone acetate plus prednisone, APA apalutamide

# Fig. S2 Plasma concentration-time profile of niraparib and its metabolite M1 at C1D28 when niraparib 200 mg was administered with apalutamide 240 mg once daily: pharmacokinetics-evaluable population


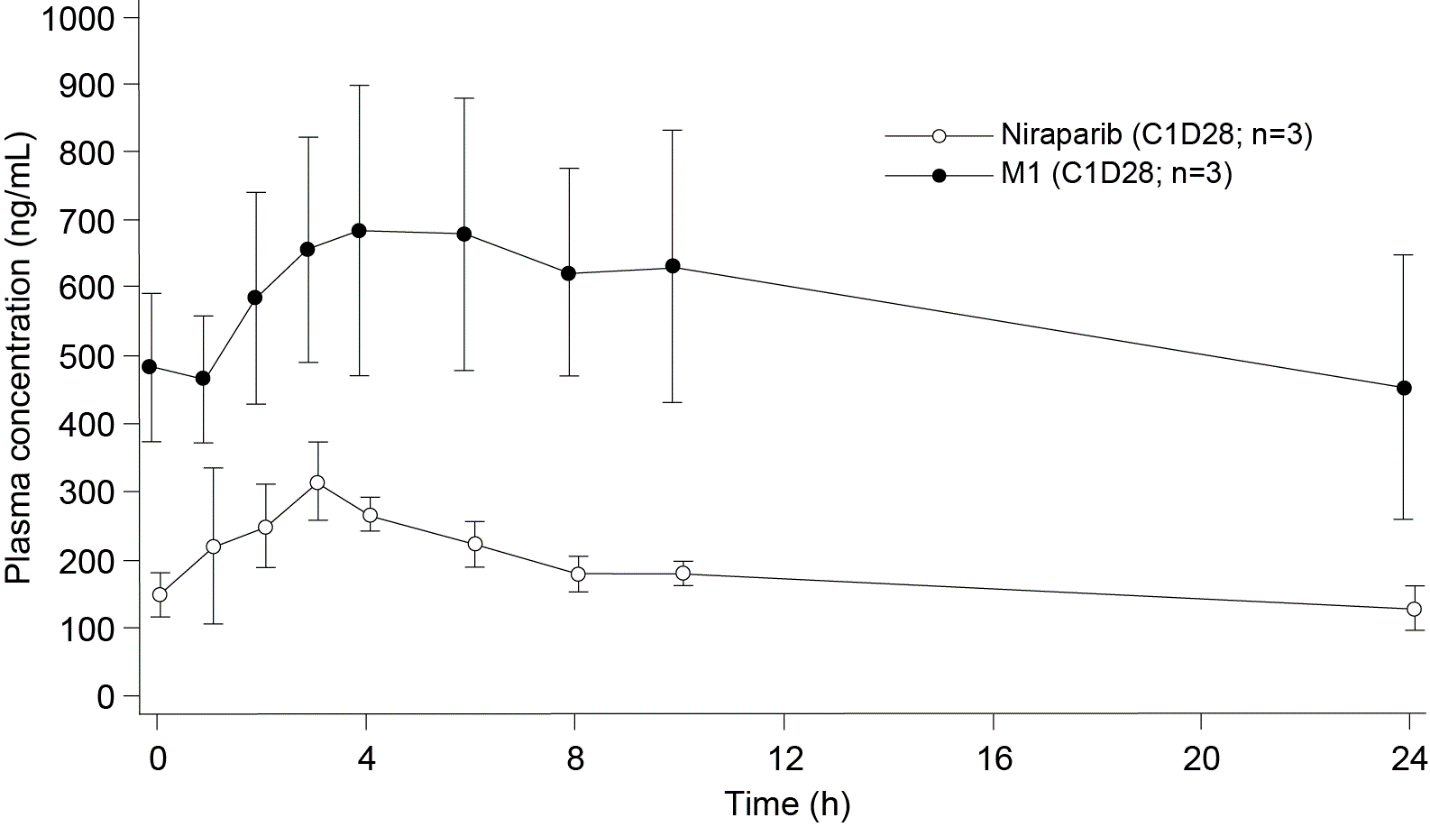


C cycle, D day

# Fig. S3 Plasma concentration-time profile of apalutamide and its metabolite M3 at C1D28 when niraparib 200 mg was administered with apalutamide 240 mg once daily: pharmacokinetics-evaluable population


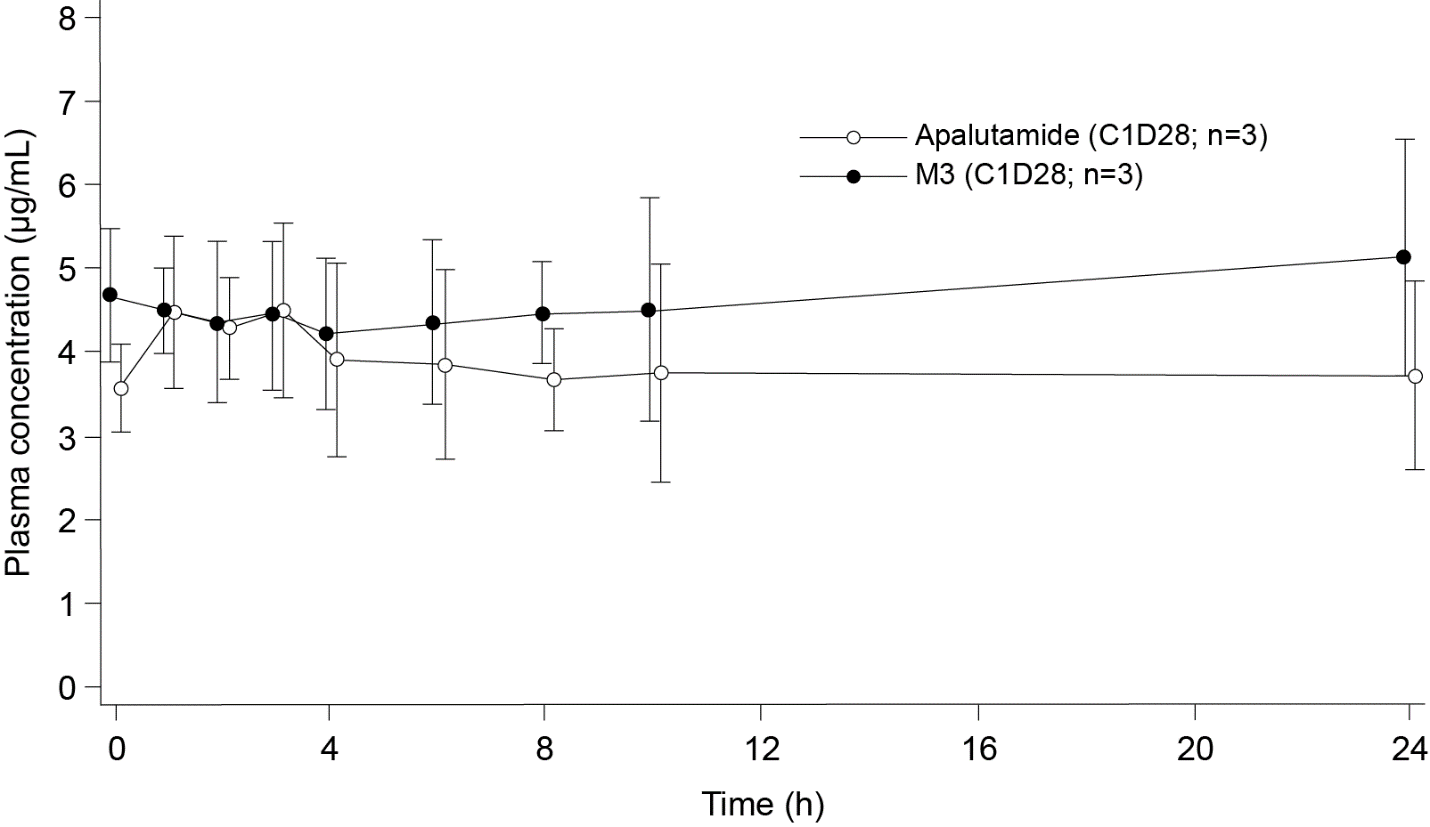


C cycle, D day

#
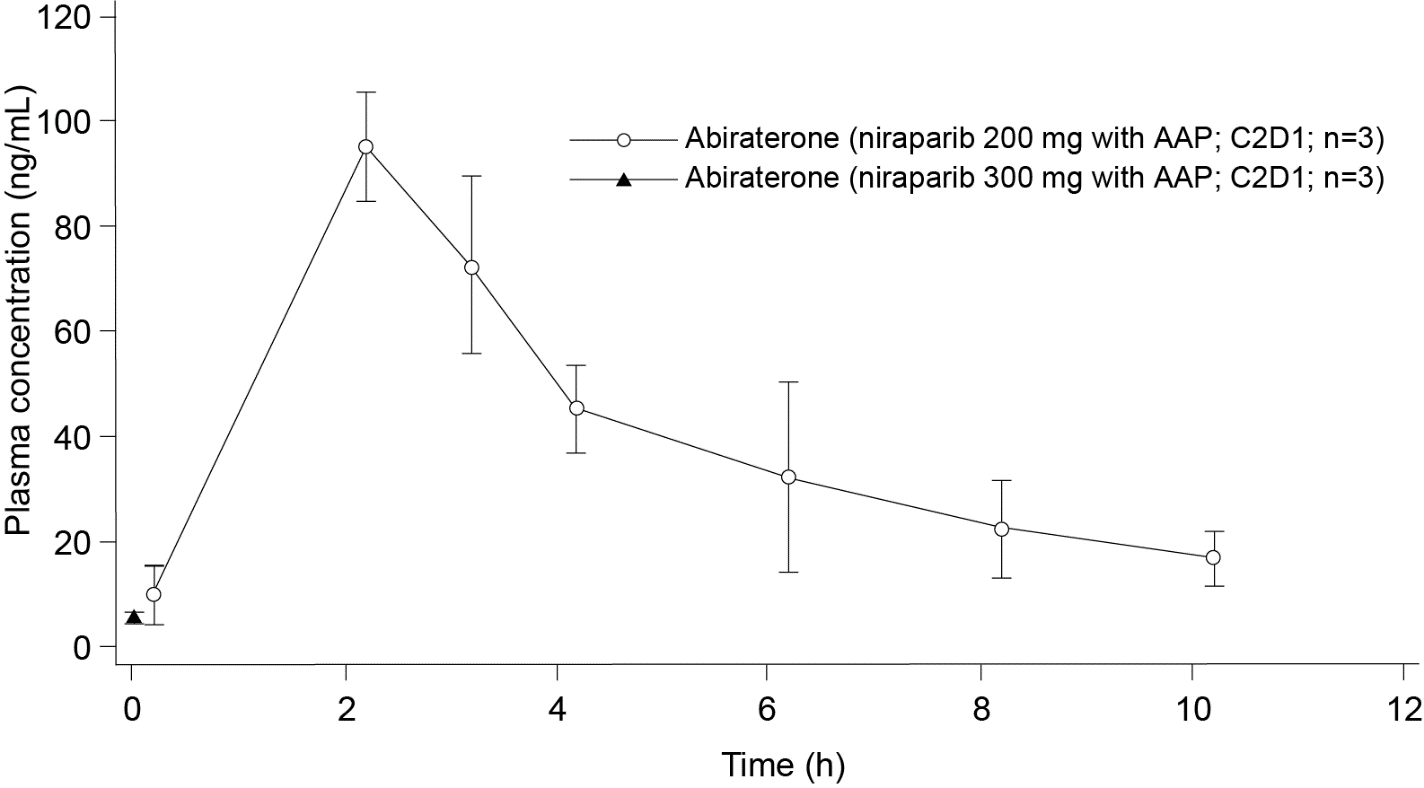
Fig. S4 Plasma concentration-time profile of abiraterone at C2D1 when niraparib 200 or 300 mg was administered with AAP once daily: pharmacokinetics-evaluable population

AAP abiraterone acetate 1000 mg with prednisone 10 mg, C cycle, D day

Note: The descriptive statistics for abiraterone with niraparib 300 mg and AAP could not be calculated because assessable data were available for only 2 patients
